# The porin VDAC2 is the mitochondrial platform for Bax retrotranslocation

**DOI:** 10.1038/srep32994

**Published:** 2016-09-13

**Authors:** Joachim Lauterwasser, Franziska Todt, Ralf M. Zerbes, Thanh Ngoc Nguyen, William Craigen, Michael Lazarou, Martin van der Laan, Frank Edlich

**Affiliations:** 1 Institute for Biochemistry and Molecular Biology, ZBMZ, Faculty of Medicine, University of Freiburg, 79104 Freiburg, Germany; 2 Faculty of Biology, University of Freiburg, 79104 Freiburg, Germany; 3 Spemann Graduate School of Biology and Medicine, SGBM, 79104 Freiburg, Germany; 4 Department of Biochemistry and Molecular Biology, Monash University, Clayton, Melbourne, Australia; 5 Department of Pediatric Medicine, Genetics, Texas Children’s Hospital, Houston, TX 77030, USA; 6 Medical Biochemistry and Molecular Biology, Saarland University, D-66421 Homburg, Germany; 7 BIOSS, Centre for Biological Signaling Studies, University of Freiburg, 79104 Freiburg, Germany

## Abstract

The pro-apoptotic Bcl-2 protein Bax can permeabilize the outer mitochondrial membrane and therefore commit human cells to apoptosis. Bax is regulated by constant translocation to the mitochondria and retrotranslocation back into the cytosol. Bax retrotranslocation depends on pro-survival Bcl-2 proteins and stabilizes inactive Bax. Here we show that Bax retrotranslocation shuttles membrane-associated and membrane-integral Bax from isolated mitochondria. We further discover the mitochondrial porin voltage-dependent anion channel 2 (VDAC2) as essential component and platform for Bax retrotranslocation. VDAC2 ensures mitochondria-specific membrane association of Bax and in the absence of VDAC2 Bax localizes towards other cell compartments. Bax retrotranslocation is also regulated by nucleotides and calcium ions, suggesting a potential role of the transport of these ions through VDAC2 in Bax retrotranslocation. Together, our results reveal the unanticipated bifunctional role of VDAC2 to target Bax specifically to the mitochondria and ensure Bax inhibition by retrotranslocation into the cytosol.

Mitochondrial apoptosis is the most common form of programmed cell death^1^ and involves proteins of the B-cell lymphoma-2 (Bcl-2) family. Bcl-2 proteins have been classified into two functional classes according to their activities in apoptosis^2^. Activation of the functionally redundant pro-apoptotic Bcl-2 proteins Bax and Bak leads to the permeabilization of the outer mitochondrial membrane (OMM) and subsequent release of intermembrane space (IMS) proteins into the cytoplasm^3^. Release of IMS proteins results in mitochondrial dysfunction and initiates the caspase cascade completely dismantling the cell^4^,^5^. Therefore Bax/Bak activation is usually the first irreversible step in intrinsic apoptosis signaling and commits the cell to apoptosis. However, recent evidence points to cellular recovery after local OMM permeabilization events^6^.

In healthy cells, pro-survival Bcl-2 proteins (e.g. Bcl-2, Bcl-x_L_ or Mcl-1) antagonize Bax and Bak by retrotranslocation from the mitochondria into the cytosol after major conformational changes^7^,^8^. Permanent translocation and retrotranslocation establish an equilibrium between cytosolic and mitochondrial Bax/Bak pools^7^,^9^,^10^. Predominant mitochondrial Bak and largely cytosolic Bax localizations result from different retrotranslocation rates caused by the hydrophobicities of the respective C-terminal transmembrane domains (TMDs)^8^. The level of mitochondrial Bax determines the cellular response to apoptosis stimulation^11^.

Apoptosis induction blocks Bax retrotranslocation and, consequently, Bax accumulates at the tips and constriction sites of mitochondria^7^,^12^,^13^. Bax activation is accompanied by conformational changes, oligomerization and insertion into the OMM^3^,^14^,^15^. Current evidence suggests that Bax, alone or in complex with other proteins, forms pores large enough to release IMS proteins^16^. Recently, ring‐like Bax structures on apoptotic mitochondria have been demonstrated using STED microscopy^17^. However, Bax has also been suggested to embed into the OMM, leading to the formation of lipidic pores also explaining IMS protein release^18^. In addition to Bax rings, Bax clusters with arc-like, line or double-line shapes have been suggested to permeabilize the OMM^19^.

On the other hand, Bax was suggested to modulate the opening of the so‐called permeability transition pore complex^20^,^21^,^22^. The voltage-dependent anion channel (VDAC), the adenine nucleotide transporter, cyclophilin D but also the F_1_F_o_ ATP synthase have been suggested to participate in this complex^20^,^21^,^22^. Whereas genetic studies seem to exclude the requirement of especially VDACs for mitochondrial apoptosis^23^, biochemical evidence points to a role of VDAC-Bax/Bak interactions in mitochondrial apoptosis signaling. VDACs are the most abundant proteins in the OMM and facilitate the transport of nucleotides, phosphocreatine, Ca^2+^ and other small ions across the OMM^24^,^25^. VDAC1 and VDAC2 are ubiquitously expressed, while VDAC3 has a more restricted organ distribution. Human VDACs share a conserved structure, forming a β-barrel across the OMM. The N-terminus has been suggested to reside inside the pore and regulate permeability^26^. Bax is present in a large VDAC2-containing complex in non-apoptotic cells^27^. VDAC2 has been suggested to inhibit Bak activation and mitochondrial apoptosis^28^, but in contrast, VDAC2-mediated Bak recruitment has been proposed as precondition for tBID-induced mitochondrial apoptosis^29^. Therefore, the role of VDAC2 in Bax/Bak-mediated mitochondrial apoptosis is currently unclear.

Here we report that VDAC2 represents the molecular platform for Bax retrotranslocation, explaining previous observations of Bax/Bak inhibition by VDAC2. However, in the absence of VDAC2 Bax fails to specifically target the OMM, suggesting a role of VDAC2 as Bax receptor in line with previous observations of a pro-apoptotic role of VDAC2 interactions with Bax/Bak. We conclude that VDAC2 has a bifunctional role to confer OMM-specific Bax recruitment and concomitant Bax inhibition via retrotranslocation.

## Results

### Bax retrotranslocation from isolated mitochondria

**Figure 1:**
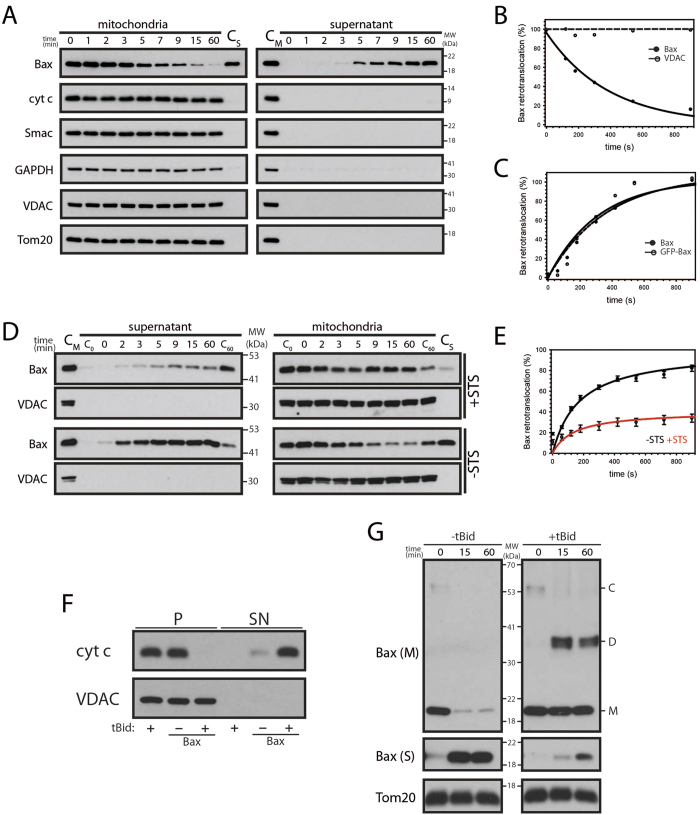
Bax retrotranslocates from isolated mitochondria. **(A)** Retrotranslocation of endogenous Bax from isolated HCT116 mitochondria is not accompanied by the movement of the intermembrane space proteins cytochrome *c* (cyt *c*) and Smac or the OMM-associated and OMM-integral proteins GAPDH, Tom20 and VDAC into the supernatant (right). The mitochondrial sample prior retrotranslocation served as control (C_M_). The retrotranslocated Bax pool in the supernatant after 60 min (C_S_) is compared to mitochondria. VDAC serves as fractionation control. n = 4. **(B)** Retrotranslocation of endogenous Bax. Remaining Bax (●) or VDAC (◯) on isolated HCT116 mitochondria relative to protein levels at 0 min. **(C)** Level of endogenous Bax (●) and GFP-Bax (◯) retrotranslocated from isolated mitochondria into the supernatant shown in Figure S1. Data is presented relative to the shuttled Bax pool at 60 min. **(D)** Endogenous Bax retrotranslocation with (top) and without (bottom) the apoptotic stimulus staurosporine (STS, 1 μM). Supernatants (left) and corresponding mitochondria (right) for indicated time points (top) with STS are compared to samples after 0 min. (C_0_) and 60 min. (C_60_) of samples without STS and vice versa. Mitochondria (C_M_) prior retrotranslocation served as control for the supernatant and supernatant after 60 min (C_S_) is compared with the mitochondria. VDAC serves as fractionation control. n = 3. **(E)** GFP-Bax retrotranslocation determined by fluorescence spectrometry in the presence (◯, red) or the absence (●, black) of STS. Bax retrotranslocation is displayed relative to the Bax shuttled in the absence of STS after 60 min. Data represent normalized averages ± SEM. n = 3. **(F)** OMM permeabilization of mitochondria isolated from HCT116 Bax/Bak DKO cells with or without endogenous Bax retrotranslocated from mitochondria (HCT116 WT cells) with or without tBid. VDAC serves as loading control. n = 3. **(G)** Cross-linking of endogenous Bax present on isolated mitochondria after incubation for up to 60 min at 37 °C in the presence of 0.5 mM DSG with and without tBid. In the absence of tBid mitochondrial Bax (Bax (M)) including a larger protein complex (C) is diminished and retrotranslocated Bax (Bax (S)) increased, while tBid induced a Bax complex (probably Bax dimers, D, roughly the size of 2x Bax monomers, M). Tom20 serves as loading control. n = 4.

The dynamic Bax shuttling between cytosol and mitochondria has been characterized by quantitative microscopy in intact cells. However, these measurements of Bax retrotranslocation required GFP-labeled protein. To extend the analysis of Bax retrotranslocation, we developed an *in organelle* retrotranslocation assay, monitoring the shuttling of Bax from purified mitochondria into the supernatant until translocation to the mitochondria and retrotranslocation from the mitochondria are in balance. Endogenous Bax retrotranslocates in a timeframe of 1 h from the mitochondria into the supernatant (Fig. 1A). During Bax shuttling the mitochondria remain intact (no Smac or cyt *c* release) and other OMM-associated and OMM-integral proteins, like GAPDH, Tom20 and VDAC, remain in the mitochondrial pellet (Fig. 1A,B). Therefore, Bax retrotranslocation specifically shuttles the Bcl-2 protein, while the integrity of isolated mitochondria is fully preserved. Endogenous Bax and GFP-Bax shuttle with similar kinetics from isolated mitochondria and the shuttling is comparable to quantitative microscopy measurements in intact cells (Figs 1C, S1, ref. 7). These results demonstrate that N-terminal GFP-fusions do not alter Bax shuttling. Furthermore Bax is retrotranslocated in the absence of cytosol. Staurosporine (STS)-induced apoptosis inhibits Bax retrotranslocation in intact cells^7^. Accordingly, Bax shuttling from mitochondria isolated from STS-pretreated cells is also inhibited (Fig. 1D,E). Of note, the kinetics of remaining Bax shuttling is comparable to the untreated control. Retrotranslocated Bax can induce OMM permeabilization in the presence of tBid (Fig. 1F). tBid-induced Bax activation and formation of Bax dimers and possibly larger oligomers largely prevents Bax retrotranslocation from isolated mitochondria (Fig. 1G). Bax retrotranslocation from isolated mitochondria is therefore comparable to Bax shuttling in intact cells.

### OMM-associated and –integral Bax retrotranslocates

**Figure 2:**
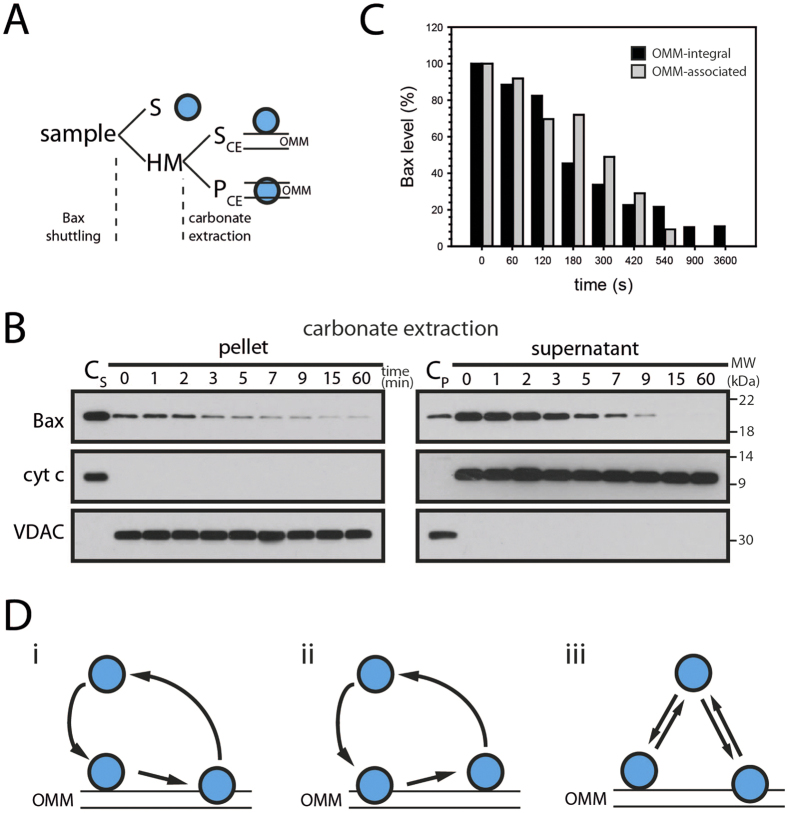
Membrane-integral Bax retrotranslocates. **(A)** The analysis of different Bax pools after retrotranslocation involves the separation of mitochondria-containing heavy membrane fraction (HM) and supernatant (S), as in Fig. 1. Outer mitochondrial membrane (OMM)-integral and OMM-associated Bax (blue, right) are further separated by carbonate extraction of the HM fraction into supernatant (S_C_, containing OMM-associated proteins) and pellet (P_C_, OMM-integral proteins), as in **B**). **(B)** Retrotranslocation of OMM-integral (left) and corresponding OMM-associated (right) endogenous Bax at indicated time points (top, in min). Supernatant (C_S_) and pellet (C_P_) of carbonate extraction prior to Bax retrotranslocation serve as controls and the fractionation is controlled by anti-cyt *c* and anti-VDAC antibodies. n = 3. **(C)** Level of Bax remaining in the OMM-associated (gray) and OMM-integral fractions (black) after indicated time points of Bax retrotranslocation. **(D)** Bax (blue) retrotranslocation from the outer mitochondrial membrane (OMM) involves the extraction of Bax from the lipid phase of the membrane. Bax shuttling could involve only retrotranslocation of OMM-integral protein, involving a membrane-associated intermediate (i) or retrotranslocation could require the OMM-associated form of Bax (ii). Alternatively, retrotranslocation of OMM-integral and OMM-associated Bax could be independent (iii).

Retrotranslocation of Bak and Bcl-x_L_ has raised the question, whether membrane-integral protein shuttles back into the cytosol, because both Bcl-2 proteins are present in healthy cells in a considerable pool of membrane-integral protein. On the other hand the entire mitochondrial populations of Bcl-x_L_ and Bak shuttle into the cytosol^7^,^8^. The *in organelle* retrotranslocation assay allows to address this question directly by performing carbonate extraction subsequent to Bax shuttling (Fig. 2A). Figure 2B shows a considerable carbonate-inextractable Bax pool (pellet) that shuttles like membrane-associated Bax (supernatant) from isolated mitochondria. Therefore, Bax retrotranslocation involves the extraction of Bax from the lipid phase of the membrane. In fact, membrane-associated and membrane-integral Bax pools shuttle with similar kinetics from the mitochondria (Fig. 2C), indicating the involvement of both forms in the same shuttling process. Based on our insight, different scenarios involving the shuttling of OMM-integral Bax and membrane-associated Bax are possible (Fig. 2D). Our results show that the retrotranslocation of Bax and Bak^8^ or Bcl-x_L_^7^ involves the shuttling of Bcl-2 proteins with at least one OMM-integral segment into the cytosol.

### Nucleotides and divalent cations regulate Bax shuttling

**Figure 3:**
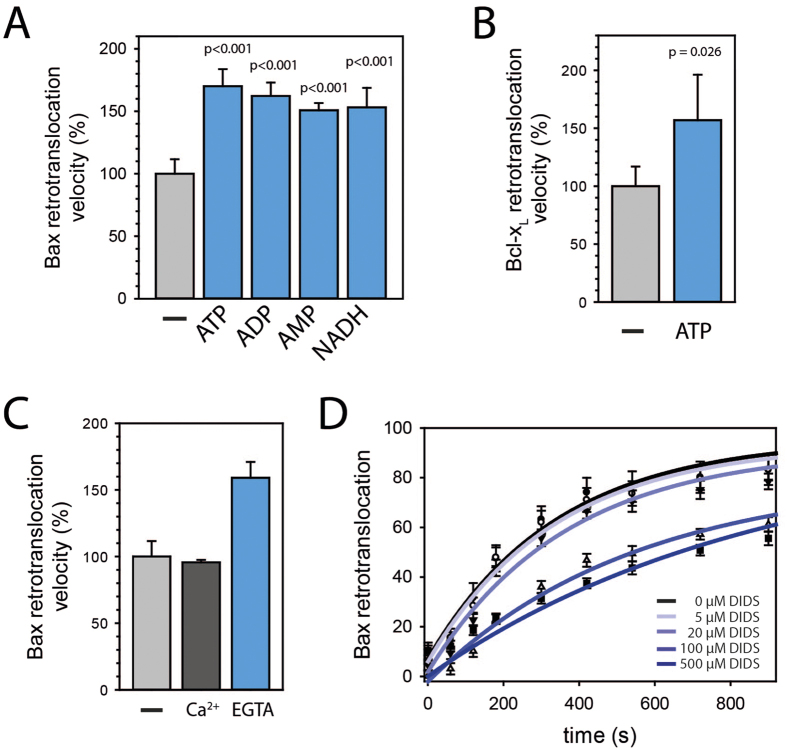
Bax retrotranslocation is regulated by nucleotides and calcium ions. **(A)** Bax retrotranslocation from isolated mitochondria in the absence (gray) and in the presence of 20 mM ATP, ADP or AMP or 10 mM NADH (blue). Data represent averages ± SEM (n ≥ 3). p-values according to One Way ANOVA are displayed. **(B)** Bcl-x_L_ shuttling from isolated mitochondria without (gray) and with ATP (20 mM, blue). Data represent averages ± SEM (n = 3). The statistically significant difference according to One Way ANOVA using Holm Sidack method is displayed. **(C)** Bax retrotranslocation from isolated mitochondria without (light gray) and with addition of Ca^2+^ (4 mM, dark gray) or EGTA (10 mM, blue) (n = 3). Data represent averages ± SEM. **(D)** GFP-Bax retrotranslocation determined by fluorescence spectrometry in the absence (●, black) or in the presence of different 4,4′-diisothiocyanostilbene-2,2′-disulfonic acid (DIDS) concentrations. Bax retrotranslocation from isolated mitochondria into the supernatant is displayed relative to the Bax shuttled in the absence of DIDS after 60 min. Data represent normalized averages ± SEM. n = 3.

We predicted that retrotranslocation of OMM-integral Bcl-2 proteins should require energy. Accordingly, the *in organelle* retrotranslocation assay demonstrated that Bax shuttling is accelerated by ATP (Fig. 3A), despite a reduction in shuttled Bax levels (data not shown). In similar manner to Bax shuttling, ATP also accelerates Bcl-x_L_ retrotranslocation from isolated mitochondria (Fig. 3A). However, faster Bax retrotranslocation was also observed in presence of ADP, AMP and NADH (Fig. 3A), suggesting a potential role of the presence of nucleotides rather than ATP hydrolysis *per se*. These results are further supported by accelerated Bax retrotranslocation in the presence of CTP, GTP or UTP (Fig. S2). ATP accelerates Bax retrotranslocation in a concentration-dependent manner (Fig. S3) and a non-hydrolysable ATP derivative accelerates Bax shuttling to the same extend as ATP (Fig. S4). Interestingly, divalent cations decrease the Bax shuttling velocity (Fig. 3c). VDACs adopt “open” and “closed” states, differing in their ability to pass non-electrolytes and to conduct ions^30^. “Closed” states are virtually impermeable to negatively charged metabolites, such as ATP, but transport small cations like Ca^2+^ 31. VDACs are thought to convert between these states by conformational changes moving a positively charged mobile domain in the wall of the channel to the surface of the membrane, which results in a pore of smaller diameter and inverted selectivity^32^. ATP binding sites have been identified in VDAC1^33^ and perhaps ATP binding induces conformational changes in VDACs. Interestingly, the VDAC inhibitor 4,4′-diisothiocyanostilbene-2,2′-disulfonic acid (DIDS) reduces Bax retrotranslocation in a concentration-dependent manner (Fig. 3D), indicating the involvement of ion transport through VDAC in Bax retrotranslocation. VDAC2 has been specifically recognized to exhibit different populations of channels with altered electrophysiological properties, suggesting cellular regulatory processes attributed specifically to VDAC2^34^. Therefore, our results indicate the possibility that Bax retrotranslocation is regulated by the transport of nucleotides and divalent cations across the OMM through different conformations of VDACs.

### VDAC2 is essential for Bax retrotranslocation

**Figure 4:**
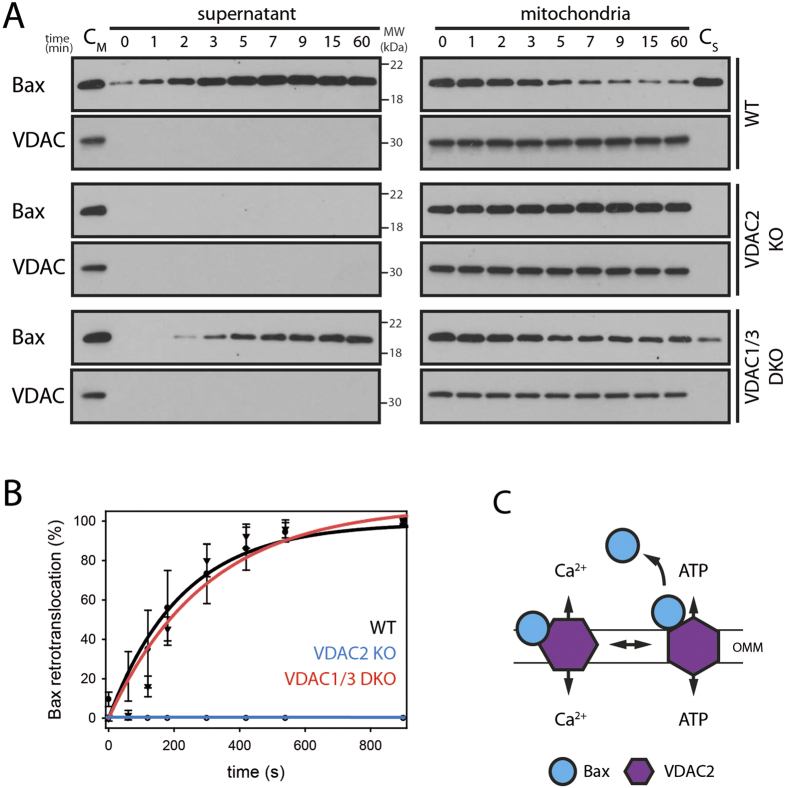
Bax fails to retrotranslocate in the absence of VDAC2. **(A)** Bax retrotranslocation from isolated mitochondria of wild-type, VDAC2 KO and VDAC1/3 DKO MEFs. Supernatants (left) and corresponding mitochondria (right) for indicated time points (top) are shown. Mitochondria prior Bax shuttling (C_M_) are compared to supernatant samples and shuttled Bax after 60 min is shown side-by-side with mitochondrial samples (C_S_). The fractionation is controlled by anti-VDAC staining. n ≥ 3. **(B)** Bax retrotranslocation from isolated mitochondria of wild-type MEFs (●, black), VDAC2 KO MEFs (◯, blue) or VDAC1/3 DKO MEFs (▼, red) relative to the shuttled Bax from wild-type MEFs after 60 min. **(C)** The shuttling of nucleotides (ATP) or calcium ions across the outer mitochondrial membrane (OMM) is thought to involve different VDAC2 (purple) conformations. Bax (blue) could bind VDAC2 in a conformation-specific manner. VDAC2 conformational changes are a potential trigger for Bax shuttling (right).

The role of porins in Bax retrotranslocation was assessed using isolated mitochondria from wild-type, VDAC2 KO and VDAC1/3 DKO mouse embryonic fibroblasts (MEFs). Bax shuttles in the wild-type and from mitochondria lacking VDAC1/3 (Fig. 4A). Strikingly, VDAC2-deficient mitochondria contained constant Bax levels, and Bax remained undetectable in the supernatant after 1 hour (Fig. 4A,B). Bax localization and shuttling from mitochondria lacking VDAC2 is also not affected by ATP (Fig. S5). Noteworthy, only the knockout of VDAC2 but not VDAC1 or VDAC3 is lethal during embryo development and conditional VDAC2 KO animals exhibit shortened lifespan and altered apoptotic responses^34^. This difference between VDAC KO cells is surprising, as VDACs have been shown to compensate for each other’s channeling activity. Our results demonstrate that Bax retrotranslocation from the mitochondria into the cytosol depends on VDAC2, indicating the possibility of Bax retrotranslocation induced by VDAC2 conformational changes^34^,^35^,^36^ (Fig. 4C).

### Bax is present in VDAC2-containing complexes on the OMM

**Figure 5:**
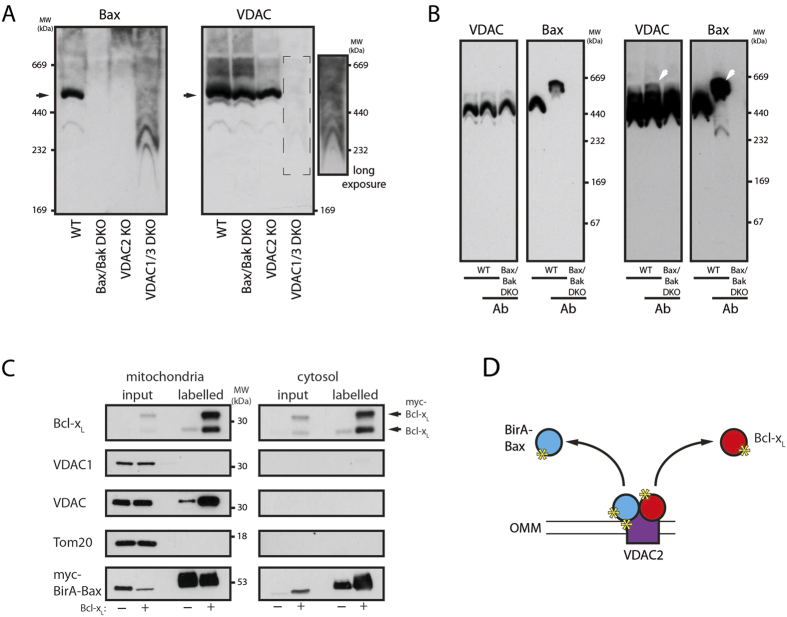
Bax and VDAC2 are present in common complexes on the OMM. **(A)** BN-PAGE analysis of isolated mitochondria from wild-type (WT), Bax/Bak DKO, VDAC2 KO (2KO) or VDAC1/3 DKO (1/3DKO) MEFs solubilized in digitonin buffer by anti-Bax (left) or anti-VDAC (right) antibodies. The apparent molecular weight (MW) of the protein complexes is indicated. VDAC-staining of VDAC1/3 DKO cells (broken line box) is shown also with a long exposure (right). Complexes containing VDAC2 and Bax are indicated by arrows on the left. n = 3. **(B)** Short (left) and long (right) exposures of the Western blot analysis of antibody induced shifts in BN-PAGE migration using anti-VDAC and anti-Bax antibodies. Bax-containing protein complexes from wild-type (WT) MEFs and samples from Bax/Bak DKO MEFs were analyzed with or without prior incubation with anti-Bax 2D2 antibody (Sigma Aldrich). Molecular weight (MW) of the protein complexes is indicated on the right. Shifted protein complexes are indicated by white arrows. n = 3. **(C)** BirA fusion assay used to identify transient Bax-interacting proteins in human cells in the absence and the presence of overexpressed Bcl-x_L_. Western blot analysis of heavy membrane (mitochondria) and cytosolic fractions using indicated antibodies reveals BirA-Bax interactions with VDAC2 and Bcl-x_L_. myc-BirA-Bax is analyzed using anti-myc antibodies. n = 3. **(D)** BirA-Bax (blue) fusion expression in human cells leads to the labeling of VDAC2 (purple) and Bcl-x_L_ (red) in close proximity to Bax with biotin (yellow asterisk) by BirA. Labelled proteins are present in the cytosolic fraction as they retrotranslocate after transient contact.

Possible interactions between Bax and VDACs were tested by blue-native (BN)-PAGE, showing a Bax-containing complex larger than 440 kDa that is absent in Bax/Bak DKO cells (Fig. 5A). This complex is also lacking in VDAC2 KO cells despite the appearance of VDAC complexes with comparable sizes, showing the requirement of VDAC2 for Bax binding. In VDAC1/3 DKO cells smaller Bax complexes appear instead of the large complex. The migration behavior of these Bax-containing complexes is very similar to that of the remaining VDAC2 complexes suggesting a reduced stability of Bax-VDAC2-containing complexes in the absence of VDAC1/3. Strikingly, Bax is predominantly present in large VDAC2-containing complexes, while significant pools of monomeric Bax or smaller Bax complexes are absent from mitochondria (Fig. S6). Specific Bax-binding to VDAC2 was corroborated by antibody shift experiments with anti-Bax antibodies shifting a minor VDAC pool (Fig. 5B). This result is expected due to the excess of VDACs compared to Bax on the OMM. The Bax TMD seems to play an important role in the formation of Bax-VDAC2-containing complexes, but intramolecular disulfide tethers constraining the exposure of the Bax BH3 domain^7^ also interfere with the formation of large Bax-containing VDAC2 complexes in favor to smaller Bax complexes (Fig. S7). Bcl-x_L_ is found in a variety of small and large complexes on the OMM that seem distinct from Bax-containing complexes. The Bcl-x_L_TBax chimera, harboring the BaxTMD instead of the corresponding Bcl-x_L_ segment at the C-terminus of Bcl-x_L_, shows again the importance of the TMD but also the cytosolic domain for complex formation on the OMM (Fig. S7). The Bcl-x_L_TBax chimera complex pattern does resemble neither that of Bcl-x_L_ complexes nor that of Bax complexes.

The interactions between Bax and VDAC2 were further investigated using a BirA fusion approach^37^. Biotin-labeling, based on transient Bax interactions as they occur during Bax retrotranslocation, is detected by subsequent purification of labelled proteins and Western blot analysis. Bax interactions were found for Bcl-x_L_ and VDAC, but not VDAC1, as shown by detection with an indiscriminative VDAC antibody and a specific VDAC1 antibody (Fig. 5C). Again, these results suggest VDAC2-Bax interactions. Labelled Bcl-x_L_ was also detected in the cytosol, despite lack of interactions between Bcl-x_L_ and Bax in the cytosol^38^,^39^. These results corroborate co-retrotranslocation of Bcl-x_L_ molecules after transiently interacting with Bax on the OMM during the retrotranslocation process (Fig. 5D). Ectopic expression of myc-Bcl-x_L_ not only shifts Bax into the cytosol but also increases the amount of labelled Bcl-x_L_ and VDAC, suggesting the more frequent interactions with Bax. These results support the role of VDAC2 as platform in Bax retrotranslocation, forming complexes with the pro-apoptotic Bcl-2 protein. Transient interactions between Bcl-x_L_ and these complexes retrotranslocate Bax.

### VDAC2 confers specific Bax-binding to the OMM

**Figure 6:**
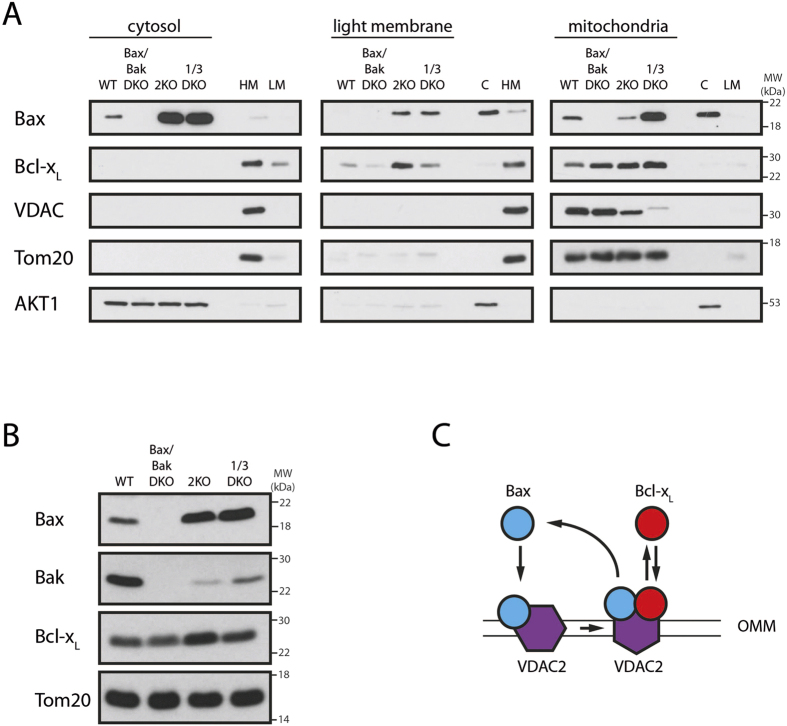
Bax specifically binds VDAC2 on the OMM. **(A)** Distribution of endogenous Bax, Bcl-x_L_ and VDAC between mitochondria-containing heavy membrane (mitochondria, right) fraction, ER-containing light membrane (center) and cytosol (left) in wild-type (WT), Bax/Bak DKO, VDAC2 KO (2KO) and VDAC1/3 DKO (1/3DKO) MEFs. Tom20 and AKT1 serve as loading controls. Fractionation is controlled by corresponding wild-type MEF fractions (HM: heavy membrane, LM: light membrane, C: cytosol). n = 3. **(B)** Western blot analysis of endogenous Bax and Bcl-x_L_ levels in wild-type, Bax/Bak DKO, VDAC2 KO and VDAC1/3 DKO MEFs. Tom20 serves as loading control. n = 3. **(C)** Bax (blue) translocation to the mitochondria targets VDAC2 (purple) in the outer mitochondrial membrane (OMM) and VDAC2 serves as platform for Bax retrotranslocation.

We next analyzed the localization of Bax in wild-type and VDAC KO cells, because VDAC2 is essential for Bax retrotranslocation. The large cytosolic Bax pool in VDAC2 KO cells compared to the wild-type is perhaps surprising given the role of VDAC2 in Bax shuttling (Fig. 6A). However, considering the presence of Bax in VDAC2-containing complexes on the mitochondria of wild-type cells, these results further support a role of VDAC2 as Bax receptor on the OMM^27^,^40^. In fact, Bax is also present in the light membrane fraction in VDAC2 KO cells, indicating the requirement of VDAC2-Bax interactions for specific OMM-targeting of Bax (Fig. 6A). Despite the importance of the formation of Bax-VDAC2-containing complexes, Bax is also present on the mitochondria of VDAC2 KO cells. Therefore, in the absence of specific targeting Bax is still competent to associate with membranes, including the OMM. In contrast to VDAC2 KO cells, VDAC1/3 DKO cells show an increased mitochondrial Bax pool, suggesting increased resistance to mitochondrial Bax under conditions when VDAC2 compensates for VDAC1/3 function. Interestingly, Bax is also present in the light membrane fraction (containing endoplasmic reticulum and other membranes) of VDAC1/3 DKO cells, suggesting a role of VDAC1/3 in the regulation of Bax association with the OMM but not Bax retrotranslocation. The shifts in Bax pools result in increased total Bax levels in VDAC2 or VDAC1/3 DKO cells, while Bak is downregulated (Fig. 6B). Cells express more Bcl-x_L_ in the absence of VDAC2 and, like Bax, Bcl-x_L_ is also present in the light membrane fraction of VDAC2 KO cells. Collectively, these results suggest that VDAC2 acts as Bax receptor and as molecular platform for Bax retrotranslocation (Fig. 6C).

## Discussion

The pro-apoptotic Bcl-2 proteins Bax and Bak are regulated by equilibriums between cytosolic and mitochondrial pools established by constant translocation to the mitochondria and retrotranslocation back into the cytosol^7^,^8^,^9^,^10^. Retrotranslocation of Bax and Bak depends on the binding of their exposed BH3 domains to the hydrophobic groove of pro-survival Bcl-2 proteins^7^,^8^. Here we identify the porin VDAC2 as essential factor for Bax retrotranslocation. Since Bax and Bak are retrotranslocated by the same process^8^, Bak retrotranslocation is likely the mechanism behind the previously reported VDAC2-mediated Bak inhibition^28^.

In addition to its central function in Bax retrotranslocation, VDAC2 is also the receptor for Bax on the OMM. Bax and Bak are present on the OMM of healthy cells in VDAC2-containing complexes with sufficient stability for native PAGE detection (Fig. 5)^27^,^40^,^41^. Our results also show direct interaction between Bax and VDAC2. Despite the role of VDAC2 and Bax-containing complexes in Bax retrotranslocation, this complex is also crucial for Bax targeting to the mitochondria of wild-type cells. Bax translocation is not mitochondria-specific in VDAC2 KO cells. Despite the pivotal role of VDAC2 in Bax binding to the OMM, VDAC1 or VDAC3 seem to play a role in stabilizing VDAC2 and Bax-containing complexes and may also facilitate Bax binding. Therefore mitochondria-specific Bax translocation is also lost in VDAC1/3 DKO cells, although VDAC2 is present. These results could explain previous co-immunoprecipitations in the presence of digitonin between VDAC1 and Bak^42^. In contrast to a previous report^27^, we find that loss of VDAC2 also affects the subcellular localization of Bcl-x_L_, suggesting a general mechanism for Bcl-2 protein targeting to the OMM. The bifunctional role of VDACs in OMM-association and retrotranslocation of Bcl-2 proteins explains contrasting findings for the function of VDACs in apoptosis regulation.

Previously the TMD of Bak has been identified to mediate interactions with VDAC2^40^. A direct interaction between Bax TMD and VDAC2 is further supported by the lack of mitochondrial targeting of Bax S184L, known to predominantly reside on wild-type mitochondria, in VDAC2 KO cells^43^. In parallel, we find that the Bax TMD is important for the formation of a common complex with VDAC2, but preventing the exposure of the Bax BH3 domain by intramolecular tethers also interfered with interactions between VDAC2 and Bax, suggesting a second binding site required for complex formation.

Bax retained the ability to bind membranes in the absence of VDAC2, as its association with artificial membranes has been widely reported^18^,^44^,^45^. However, VDAC2 is required for mitochondria-specific Bax membrane association. By recruiting Bax to the mitochondria, VDAC2 establishes an equilibrium between mitochondrial and cytosolic Bax pools quickly responding to cell stress. VDAC2-dependent shuttling adapts the level of mitochondrial Bax und thus the cellular response to apoptotic stimulation^11^. However, a second and probably equally important function of VDAC2-dependent Bax shuttling is ensuring OMM-specific Bax binding and thus prevention of Bax accumulation on different membranes in the cell.

## Methods

### Cell culture and transfection

HCT116 cells (malignant cells originally isolated from a human male with colonic carcinoma) and HCT116 Bax/Bak DKO cells with or without stable GFP-Bax expression were cultured in McCoy’s 5A medium supplemented with 10% heat-inactivated fetal bovine serum and 10 mM Hepes in 5% CO_2_ at 37 °C. MEFs were cultured in DMEM supplemented with 10% heat-inactivated fetal bovine serum and 10 mM Hepes in 5% CO_2_ at 37 °C. Cells were transfected with Turbofect (Fermentas) or Lipofectamine LTX (Invitrogen), typically with 100 ng of Bax or Bcl-x_L_ constructs in pEGFP vector according to the manufacturer’s instructions.

### Whole cell lysis and subcellular fractionation

Cells were harvested and incubated in cell lysis buffer (10 mM Hepes pH7.4, 150 mM NaCl, 1% Triton X100, protease inhibitor mix) for 15 min on ice. Whole cell extracts were obtained by centrifugation at 15.000 × g for 10 min at 4 °C. Samples were boiled in SDS-sample buffer for 5 min at 95 °C and subsequently subjected to SDS-PAGE and western blot analysis.

Subcellular fractionations into cytosolic and heavy membrane fractions were performed as previously described^11^. Cells were harvested with PBS, centrifuged at 1,500 × g for 5 min, washed in PBS and resuspended in SEM buffer (10 mM HEPES, 250 mM sucrose, pH 7.2) supplemented with protease inhibitors. After 10 min incubation on ice, cells were broken by two rounds of 10 passages through a 25G needle (Braun) fitted on a 1 ml syringe (Braun) in the absence of detergents. After each round the lysate was cleared from debris and unbroken cells by centrifugation for 5 min at 1,000 × g and the supernatant of both steps was collected and pooled. The suspension was centrifuged at 8,000 × g at 4 °C for 10 min. The resulting pellet, representing the heavy membrane fraction, was washed one time in SEM buffer and one time in KCl buffer (125 mM KCl, 4 mM MgCl_2_, 5 mM Na_2_HPO_4_, 5 mM succinate, 0.5 mM EGTA, 15 mM HEPES–KOH, pH 7.4) supplemented with protease inhibitors. The supernatant was first centrifuged at 15,000 × g at 4 °C for 10 min and the resulting supernatant centrifuged again at 150,000 × g at 4 °C for 30 min to separate the cytosolic fraction (supernatant) from the light membrane fraction (pellet, containing endoplasmic reticulum, GOLGI, peroxisomes and other membrane systems). The ultracentrifugation pellet was rinsed once with SEM buffer. The cytosolic fraction was acetone-precipitated and the pellets of all fractions boiled for 5 min at 95 °C in SDS sample buffer and applied to SDS-PAGE and western blot analysis.

### Analysis of native protein complexes

To analyze native protein complexes, isolated mitochondria were solubilized in digitonin buffer (20 mM Tris-HCl, pH 7.4, 50 mM NaCl, 0.1 mM EDTA, 10% [v/v] glycerol, 2% [w/v] digitonin, 1 mM PMSF) and subsequently incubated at 4 °C for 45 min in an overhead shaker. After insoluble material was removed by centrifugation (16,000 × g, 10 min, 4 °C) mitochondrial extracts were subjected to blue native PAGE^46^ using 4–13% continuous polyacrylamide gradient gels.

### *In organelle* retrotranslocation assay

Subcellular fractionation was conducted as described above. In cases were cells have been treated with substances, all buffers used during the fractionation have been supplemented with these substances accordingly. Purified mitochondria were resuspended in KCl buffer supplemented with protease inhibitors and indicated substances and aliquoted to one sample per time point for western blot analysis and two samples per time point for fluorometric measurements. After incubation at 37 °C for the indicated time, each sample was put on ice shortly and separated into mitochondrial pellet and supernatant by centrifugation for 5 min at 10,000 × g at 4 °C. For western blot analysis mitochondrial pellets were washed once with KCl buffer while supernatant samples were acetone precipitated. The resulting pellets of both fractions were boiled in SDS sample buffer and subjected to SDS-PAGE and western blot.

GFP-tagged BAX was measured in duplicates by fluorometry, using a PerkinElmer Victor X plate reader with excitation filters at 485 nm and emission filters at 535 nm wavelengths. Mitochondrial pellets were resuspended in KCl-buffer containing 1% (v/v) Triton X100 and centrifuged for 5 min at 10,000 × g at 4 °C prior measurement.

### Carbonate extraction

After conducting a subcellular fractionation or an in organelle retrotranslocation assay as described above, the resulting mitochondrial pellets were washed two times in SEM buffer and resuspended in SEM buffer supplemented with 100 mM Na_2_CO_3_ and protease inhibitors, adjusted to pH 11.5. The samples were incubated on ice for 30 min. Then the extract was centrifuged at 140.000 × g for 30 min at 4 °C. The supernatant, carbonate extractable proteins, was subjected to protein precipitation by trichloroacetate (TCA). The pellet, containing carbonate inextractable proteins, was washed once more in SEM buffer supplemented with 100 mM Na_2_CO_3_ and protease inhibitors, adjusted to pH 11.5. Both fractions were assayed by SDS-PAGE and western blot.

### BirA assay

HCT116 cells were transfected with pcDNA3-mycBioID-Bax plasmid, resulting in myc-tagged BirA-Bax-fusion expression. After cell harvest in ice-cold PBS, the cell pellet was resuspended and lysed in SEM buffer (10 mM Hepes, pH 7.2, 250 mM sucrose, complete proteinase inhibitor cocktail (Roche) and 0.2% Triton X100). Cell lysate was centrifuged (120,000 × g, 30 min, 4 °C) and washed using a concentrator column (Vivaspin, GE Healthcare, 100 mM Tris-HCl, pH 8.0, 150 mM NaCl, 5 mM EDTA, 0.1% Triton X100, complete proteinase inhibitor cocktail (Roche)). Then input-sample (2.5%) was separated and the remaining lysate incubated with streptavidin agarose beads (Thermo, O.N., 4 °C). Subsequently, beads were washed and boiled in SDS-sample buffer. Input and samples were analyzed by SDS-PAGE and western blot.

### Crosslinking experiments

After incubation of mitochondria at 37 °C for indicated time periods, pellet and supernatant fractions were subjected to crosslinking for 1 h at 4 °C using 0.5 mM DSG. Samples were precipitated and analyzed by Western Blot.

### Cyt *c* release from isolated mitochondria

Mitochondria (100 µg) isolated from HCT116 Bax/Bak DKO cells were incubated in the absence and the presence of Bax retrotranslocated from mitochondria isolated from wildtype HCT116 cells for 15 min at 30 °C. OMM permeabilization was induced with recombinant tBid (R&D Systems, 10 nM) for 1 h at 37 °C. The mitochondria were then centrifuged for 5 min at 13,000 g at 4 °C. Mitochondrial pellets corresponding to 10 µg of protein and the corresponding volume of supernatant fractions were resolved by SDS–PAGE and transferred to a nitrocellulose membrane. VDAC serves as control.

## Additional Information

**How to cite this article**: Lauterwasser, J. *et al*. The porin VDAC2 is the mitochondrial platform for Bax retrotranslocation. *Sci. Rep.* **6**, 32994; doi: 10.1038/srep32994 (2016).

## Supplementary Material

Supplementary Information
