# Supplementary material for: The porin VDAC2 is the mitochondrial platform for Bax retrotranslocation
Source: Sci Rep. 2016 Sep 13;6:32994. doi: 10.1038/srep32994 (PMC5020405; doi:10.1038/srep32994)
Supplement: Supplementary Information [file srep32994-s1.pdf]

The porin VDAC2 is the mitochondrial platform for Bax retrotranslocation

Joachim Lauterwasser[1,4], Franziska Todt[1], Ralf M. Zerbes[1,4], Tran Nguyen[5], William Craigen[6], Michael Lazarou[5], Martin van der Laan[1,2,7] and Frank Edlich[1,2,*]

[1]Institute for Biochemistry and Molecular Biology, ZBMZ, Faculty of Medicine, University of Freiburg, 79104 Freiburg, Germany

[2]BIOSS, Centre for Biological Signaling Studies, University of Freiburg, 79104 Freiburg, Germany

[3]Spemann Graduate School of Biology and Medicine, SGBM, 79104 Freiburg, Germany

[4]Faculty of Biology, University of Freiburg, 79104 Freiburg, Germany

[5]Department of Biochemistry and Molecular Biology, Monash University, Clayton, Melbourne, Australia

[6]Department of Pediatric Medicine, Genetics, Texas Children's Hospital, Houston, TX 77030

[7]Medical Biochemistry and Molecular Biology, Saarland University, D-66421 Homburg, Germany

*Contact: F. Edlich, frank.edlich@biochemie.uni-freiburg.de

Short title: VDAC2 is essential for Bax retrotranslocation

Key words: Bcl-2 proteins, mitochondrial apoptosis, voltage-dependent anion channels, mitochondria

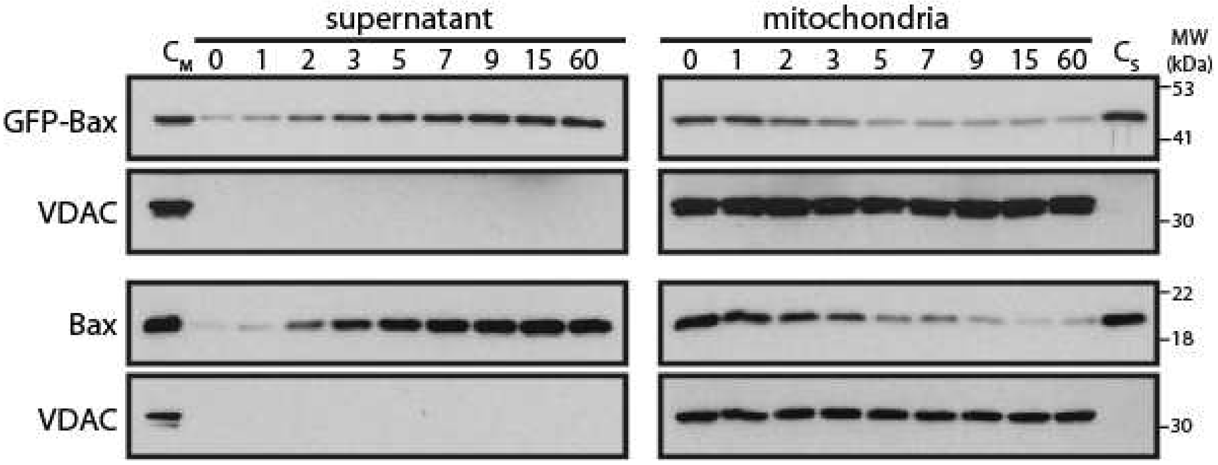

Supplementary Figure S1. Retrotranslocation of endogenous Bax (bottom panels) and GFP-Bax (top panels) from isolated mitochondria at indicated time points (in min.) analyzed by Western blot. Mitochondria prior to retrotranslocation served as control (C_M). The corresponding mitochondrial fractions at indicated time points are shown on the right compared to shuttled Bax after 60 min. (C_S). VDAC serves as fractionation control. n = 3.

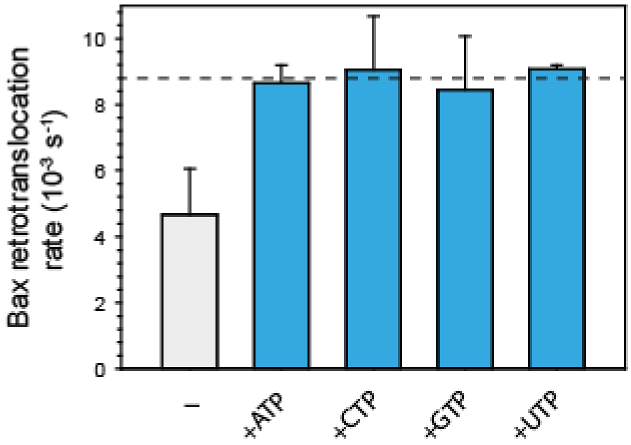

Supplementary Figure S2. Bax retrotranslocation from isolated mitochondria in the absence (gray) and in the presence of 10 mM ATP, CTP, GTP and UTP (blue). Data represent averages ± SEM (n ≥ 3). The broken shows the Bax retrotranslocation rate determined in cells in the presence of ectopic Bcl-x_L expression.

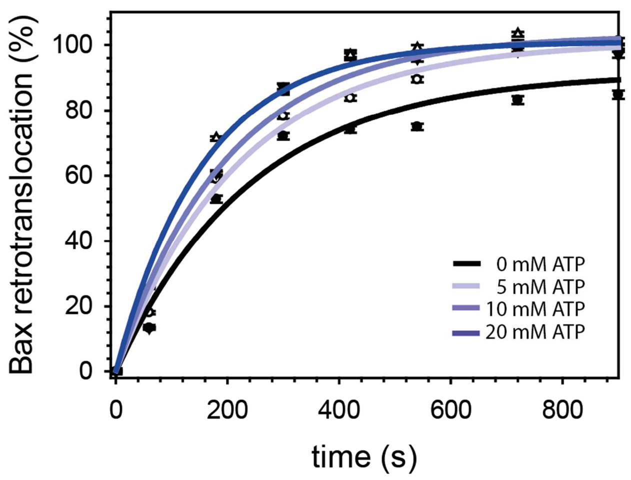

Supplementary Figure S3. Effect of different ATP concentrations (0 mM ATP: black line, ●; 5 mM ATP: light blue line, ○; 10 mM ATP: blue line, ▲; 20 mM ATP; dark blue line, △) on GFP-Bax retrotranslocation from isolated mitochondria into the supernatant. Data is presented as averages ± SEM relative to the shuttled Bax pool at 60 min.

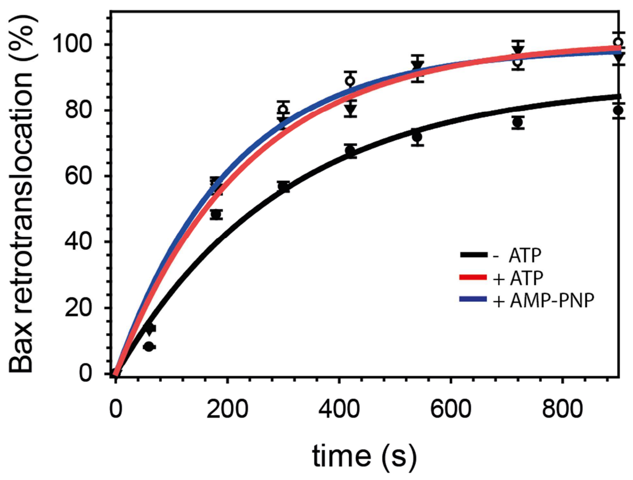

Supplementary Figure S4. Acceleration of GFP-Bax retrotranslocation in the absence (black line, ●) or the presence of 10 mM ATP (red line, ○) or 10 mM non-hydrolysable AMP-PNP (blue line, ▲) from isolated mitochondria into the supernatant. Data is presented as averages ± SEM relative to the shuttled Bax pool at 60 min.

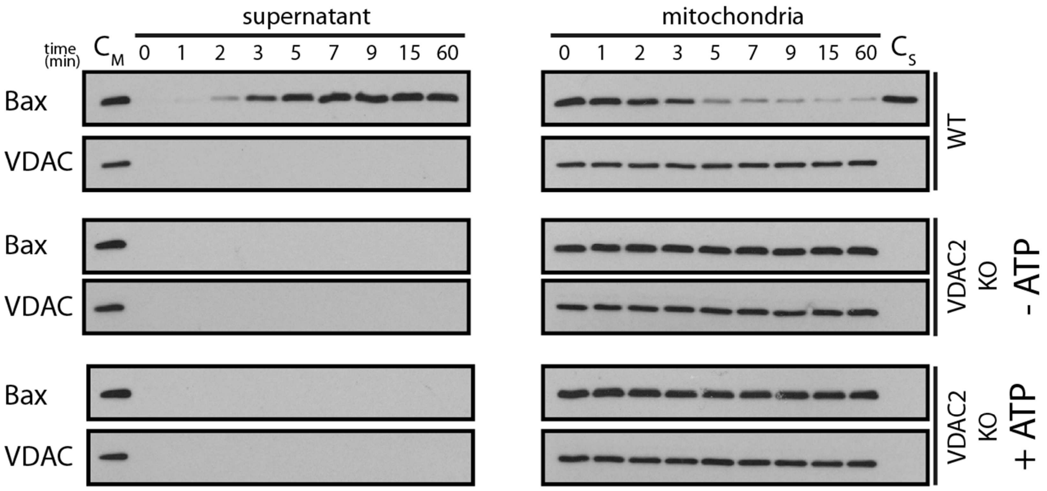

Supplementary Figure S5. Bax retrotranslocation from isolated mitochondria of wild-type and VDAC2 KO MEFs in the absence and the presence of 10 mM ATP. Supernatants (left) and corresponding mitochondria (right) for indicated time points (top) are shown. Mitochondria prior Bax shuttling are compared to supernatant samples and shuttled Bax after 60 min (C_M). Supernatant is shown side-by-side with mitochondrial samples (C_S). The fractionation is controlled by anti-VDAC staining. n ≥ 3.

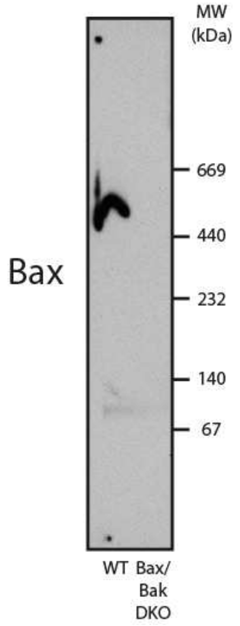

Supplementary Figure S6. Western blot analysis of large protein complexes residing on wild-type and Bax/Bak DKO MEF mitochondria solubilized in digitonin-containing buffer by BN-PAGE using anti-GFP antibodies. Apparent molecular weight (MW) of the protein complexes is indicated on the right.

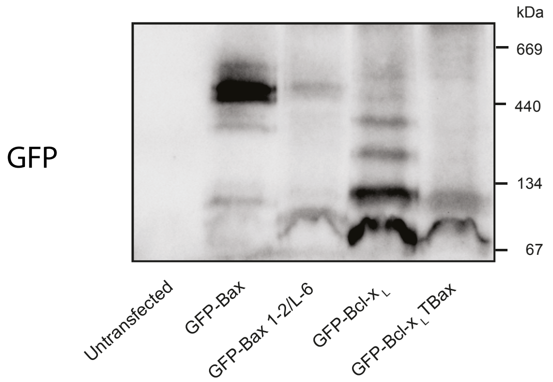

Supplementary Figure S7. Western blot analysis of large protein complexes residing on mitochondria solubilized in digitonin-containing buffer by BN-PAGE using anti-GFP antibodies. HCT116 Bax/Bak DKO cells expressed wild-type GFP-Bax, GFP-Bax 1-2/L-6 (constraining the conformation of the Bax BH3 (Edlich et al., 2011)), GFP-Bcl-x_L or the chimera GFP-Bcl-x_LTBax (Bcl-x_L TMD substituted by the corresponding Bax TMD (Todt et al., 2013)). Apparent molecular weight (MW) of the protein complexes is indicated on the right.
